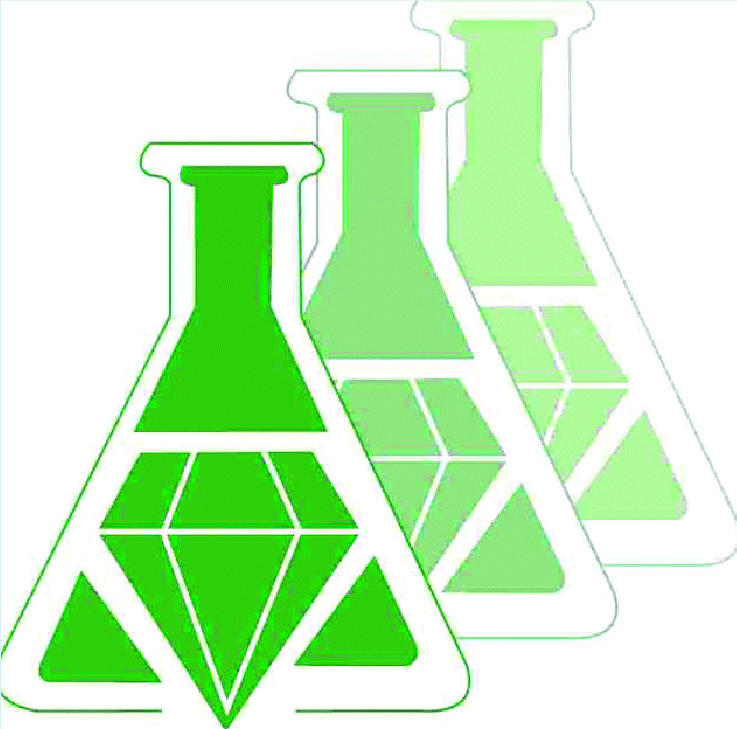# EHPnet: Greener Education Materials for Chemists

**Published:** 2005-11

**Authors:** Erin E. Dooley

Green chemistry aims in part to help clean up chemical processing by reducing or eliminating toxic elements from production and use. One university at the forefront of the movement is the University of Oregon, which has developed a website, Greener Education Materials for Chemists (GEMS), to educate teachers on introducing green chemistry concepts to their students. Although the site, located at **http://greenchem.uoregon.edu/gems.html**, currently contains only materials for university-level education, the developers hope to eventually include content for K–12 teachers.

The site consists of a database of print resources, which visitors can search using free text or by selecting search terms from seven categories, including chemistry concepts, laboratory techniques, green chemistry principles, and chemistry sub-disciplines. Each item in the database has an overview that summarizes its content and its connection to green chemistry as well as contact information for the person that contributed the material to the database.

The different types of material that are currently available on the site, which is partially funded by the National Science Foundation, include laboratory exercises, lecture materials, course syllabi, and multimedia content. To aid educators in determining which materials best suit their needs, threaded discussions will soon be included for each item. Here educators will be able to discuss how they integrated materials into their lesson plans and relate their success in using them.

The site, unveiled in June 2005 at an American Chemical Society meeting, was developed by a partnership between the university’s Green Chemistry Group and Center for Educational Technologies. Students and high school teachers were involved in the design of the site, as were more than 100 college instructors who attended national green chemistry education workshops at the university. The site’s developers have provided information on the site advising people how to contribute material to the database. They are also looking for educators to evaluate materials, test laboratory procedures, and adapt content for varying age groups. The developers want the web-site to be as inclusive as possible so it can serve as many grade levels and subject areas as possible.

A link to information about the university’s Green Chemistry Center is sited in the toolbar at the top of the homepage. Here visitors can find an overview of the program’s work in developing undergraduate green chemistry curricula, the history of the program, and media coverage. A description of *Green Organic Chemistry: Tools, Strategies and Laboratory Experiments*, a textbook/laboratory manual released in 2004 for the undergraduate organic chemistry laboratory, is available from this page as well.

## Figures and Tables

**Figure:**